# Vitamin D status and asthma, lung function, and hospitalization among British adults

**DOI:** 10.3389/fnut.2022.954768

**Published:** 2022-08-10

**Authors:** Yiqun Zhu, Danrong Jing, Huaying Liang, Dianwu Li, Qinyu Chang, Minxue Shen, Pinhua Pan, Hong Liu, Yan Zhang

**Affiliations:** ^1^Center of Respiratory Medicine, Xiangya Hospital, Central South University, Changsha, China; ^2^National Clinical Research Center for Geriatric Disorders, Xiangya Hospital, Central South University, Changsha, China; ^3^Hunan Engineering Research Center for Intelligent Diagnosis and Treatment of Respiratory Disease, Changsha, China; ^4^Department of Social Medicine and Health Management, Xiangya School of Public Health, Central South University, Changsha, China; ^5^National Key Clinical Specialty, Branch of National Clinical Research Center for Respiratory Disease, Xiangya Hospital, Central South University, Changsha, China; ^6^Department of Dermatology, Xiangya Hospital, Central South University, Changsha, China

**Keywords:** vitamin D, asthma, lung functions, wheeze, UK Biobank

## Abstract

**Background:**

Vitamin D has been known to be associated with asthma. However, the association between vitamin D status and asthma, lung function as well as hospitalization among adults remains unclear.

**Objective:**

To investigate the role of serum vitamin D in asthma prevalence, lung function, and asthma control in adults.

**Methods:**

Multivariable logistic regression was applied to assess the relationship between serum vitamin D and asthma prevalence, lung function (FEV1, FVC, and FEV1/FVC), current wheeze, and asthma-linked hospitalizations in a cross-sectional study of 435,040 adults aged 37–73 years old from the UK Biobank.

**Results:**

Compared to vitamin D deficiency, the odds of asthma were decreased by 6.4% [adjusted odds ratio (*aOR*) = 0.936; 95% *CI*: 0.911–0.962; *p* < 0.001] and 9.8% (*aOR* = 0. 0.902; 95% *CI*: 0.877–0. 0.927; *p* < 0.001) in individuals with insufficient and optimal vitamin D concentration, respectively, in the fully adjusted model. In total asthmatic patients, serum vitamin D was obviously and positively related with FEV1 (β = 1.328 ml, 95% *CI* = 0.575–2.080), FVC (β = 2.018 ml, 95% *CI* = 1.127–2.908), and FEV1/FVC (β = 0.006%, 95% *CI* = 0.002–0.010). Asthmatic patients whose vitamin D level was in the deficient category had 9.3–19.9% higher odds of current wheeze than insufficient categories (*aOR* = 0.907; 95% *CI*: 0.861–0.957; *p* < 0.001) and optimal categories (*aOR* = 0.801; 95% *CI*: 0.759–0.845; *p* < 0.001), but the relationship between vitamin D and asthma hospitalization was not significant.

**Conclusion:**

Vitamin D deficiency was related to higher odds of asthma and current wheeze, and lower lung function in a large sample size study of British adults. Our results indicate a potential positive impact of serum vitamin D on asthma occurrence and disease control in adults.

## Introduction

Asthma is one of the most prevailing chronic airway diseases all over the world and affects all age groups, such as more than 300 million people worldwide (1, 2). An epidemiological investigation estimated the global burden of doctor-diagnosed asthma in adults among 70 participating countries at 4.3% (3). Among the whole enrolled countries, the lowest prevalence was 0.2% in China while the highest was 21.0% in Australia (3). Although effective preventive measures are available, costs connected with asthma still increase, consisting of direct and indirect costs (4). Asthma prevention, as a major public health priority, should be emphasized.

It is well known that vitamin D, easily assessed by serum vitamin D levels, can regulate the homeostasis of calcium and phosphorus and adjust bone metabolism. In humans, the supplement of vitamin D mostly comes from sun exposure (5). Vitamin D deficiency could be found in some chronic diseases, such as rheumatoid arthritis, diabetes, inflammatory bowel disease, and asthma (6–10). The co-existence was partly explained by the immunomodulatory effect of Vitamin D. Vitamin D effects through binding with the vitamin D receptor (VDR) and then acted on the host of immune cells, such as macrophages, T and B lymphocytes, dendritic cells (DCs), as well as structural cells in the airways (11, 12).

To date, great interest in the effect of vitamin D in the occurrence, development, and treatment of asthma has been increasing and extensive observational studies have been performed with conflicting results. Some studies demonstrated that a lower level of vitamin D was associated with higher asthma prevalence (13, 14) while other studies found no association (15–17). Some studies reported the association of vitamin D and asthma control (17–19). Meanwhile, the previous studies have mostly focused on children; the evidence among adults is limited and inconclusive.

Given discrepant findings for a possible role of vitamin D in asthma from diverse studies, we explored the relationship between serum vitamin D and asthma, lung function, current wheeze, and asthma-related hospitalizations in a large cohort study from UK Biobank.

## Materials and methods

### Study design and participant

More than 500,000 participants aged between 37 and 73 were recruited for the UK Biobank project with its unique sample size and scope (20). An electronic signed consent brief computer-assisted interview, self-completed touch-screen questionnaire, functional measures, physical examination, and collection of urine, blood, and saliva were included (21). The current study was based on the data of the UK Biobank that has received approval from the North West Multi-Center Research Ethics Committee and all individuals included signed informed consent (Application Number 84,979). The UK Biobank website shows more information about the process, definitions, and so on.^1^ Participants with missing data or extreme value of vitamin D, and individuals without crucial covariates including age, sex, body mass index (BMI), and smoking status were also excluded.

### Definition

Participants were asked the question: “Has a doctor ever told you that you have had any of the following situations: hay fever, cystic fibrosis, asthma, chronic obstructive pulmonary disease (COPD), allergic rhinitis, emphysema, chronic bronchitis, and so on?” Asthma was one of the choices. Individuals who had current wheeze answered “Yes” to the question: “In the last year have you ever had wheeze or whistling in the chest?” Hospitalization for asthma was defined as ever having had a hospitalization with a main diagnosis compatible with asthma, excluding hospitalizations with a main diagnosis consistent with COPD. The International Classification of Diseases Clinical Modification (ICD) code for asthma included ICD-9: 493.x or ICD-10: J45.x and J46.x, while ICD code for COPD consisted of ICD-9: J43, J44, J47 or ICD-10: 490, 491, 492, 494, 496.

### Measurements

The detailed information of the measurements was provided on the UK Biobank. The UK Biobank has collected biological samples of all 500,000 participants and measured a series of biochemical markers at baseline. The concentration of 25(OH)D was measured in a range from 10 to 375 nmol/L. Serum vitamin D level was determined by a direct competitive Chemiluminescent Immunoassay. A series of robust and comprehensive quality procedures was established to reduce drift, bias, and measurement uncertainty. A rigorous external quality assurance (EQA) and internal quality control (QC) protocol guided the evaluation of biomarkers. The lung function test is not undertaken if participants were pregnant (1st or 3rd trimester), being treated for tuberculosis, had history of a collapsed lung, a detached retina, had a chest infection in the last month or a heart attack, eye surgery, chest, or abdominal surgery in the previous 3 months. Lung function measurements were recorded using a spirometer (Vitalograph Pneumotrac 6800) with two or three blows and were guaranteed to be acceptable and reproducible. The good reproducibility of the first two blows was defined as a < 5% difference in FVC and FEV1, and the third blow was deemed unnecessary. Investigators determined whether the spirometry was adequate and acceptable.

### Statistical analysis

**FIGURE 1:**
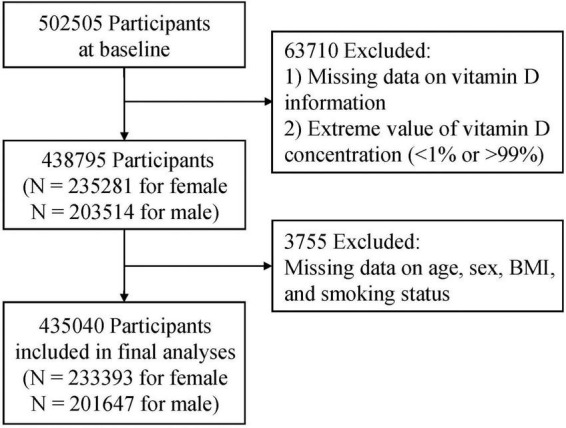
Flow chart of the study population.

To get bivariate analyses, *t*-test or chi-square tests were used appropriately. Meanwhile, logistic regression was applied to get the multivariable analyses of serum vitamin D and asthma prevalence, current wheeze, asthma hospitalization, and lung function. Adjusting sex, age, ethnicity, BMI, income, smoking status, education, and vitamin D supplements for all models. Models for FEV1 and FVC were additionally adjusted for height and height squared. Due to the missing data for covariates, 3,755 of the available individuals were excluded from the analysis (Figure 1), but the sensitivity analysis included those participants by multiple imputation procedure.

## Results

In the current study, 435,040 participants were included in the final analysis after excluding 63,710 participants with missing data on vitamin D or extreme value of vitamin D concentration (<1% or >99%), and 3,755 cases without data on age, sex, BMI, and smoking status (Figure 1).

The major characteristics of the included participants grouped by asthma status were presented in Table 1. Of the 435,040 participants, 58,635 had doctor-diagnosed asthma (hereafter called “asthma”). In contrast to control subjects (*n* = 376,405), asthmatic patients were more likely to be female, younger, and person with poorer education and lower income, and to have higher BMI but lower vitamin D status and lower lung function (FEV1, FVC, and FEV1/FVC).

**TABLE 1:** Characteristics of the included participants from the UK Biobank.

Covariate	Total (*N* = 435,040)	Asthma (*N* = 58,635)	No asthma (*N* = 376,405)	*P*
Age at baseline (years), Mean (*SD*)	56.50 (8.11)	56.13 (8.27)	56.56 (8.09)	< 0.001
Age category (years), *N* (%)				< 0.001
<50	102,949 (23.7)	15,105 (25.8)	87,844 (23.3)	
50–59	144,179 (33.1)	19,133 (32.6)	125,046 (33.2)	
> 60	187,912 (43.2)	24,397 (41.6)	163,515 (43.5)	
Sex, *N* (%)				< 0.001
Female	233,393 (53.6)	33,371 (56.9)	200,022 (53.1)	
Male	201,647 (46.4)	25,264 (43.1)	176,383 (46.9)	
Ethnicity, *N* (%)				< 0.001
White	395,084 (90.8)	53,376 (91.0)	341,708 (90.8)	
Mixed	15,895 (3.7)	2,150 (3.7)	13,745 (3.7)	
Asian or Asian British	15,163 (3.5)	1,917 (3.3)	13,246 (3.5)	
Black or Black British	2,348 (0.5)	357 (0.6)	1,991 (0.5)	
Chinese	1,364 (0.3)	134 (0.2)	1,230 (0.3)	
Other ethnic groups	3,726 (0.9)	493 (0.8)	3,233 (0.9)	
Unknown	1,460 (0.3)	208 (0.4)	1,252 (0.3)	
Average total household income before tax (€), *N* (%)				< 0.001
Less than 18,000	83,214 (19.1)	12,644 (21.6)	70,570 (18.7)	
18,000–30,999	94,586 (21.7)	12,277 (20.9)	82,309 (21.9)	
31,000–51,999	97,873 (22.5)	12,544 (21.4)	85,329 (22.7)	
52,000–100,000	77,067 (17.7)	9,992 (17.0)	67,075 (17.8)	
Greater than 100,000	20,583 (4.7)	2,662 (4.5)	17,921 (4.8)	
Unknown	61,717 (14.2)	8,516 (14.5)	53,201 (14.1)	
Education, *N* (%)				< 0.001
College or university degree	142,036 (32.6)	19,049 (32.5)	122,987 (32.7)	
Professional qualifications	51,300 (11.8)	6,881 (11.7)	44,419 (11.8)	
A Levels/AS Levels or equivalent	48,534 (11.2)	6,523 (11.1)	42,011 (11.2)	
O Levels/GCSEs or equivalent	115,671 (26.6)	14,990 (25.6)	100,681 (26.7)	
None of the above	77,499 (17.8)	11,192 (19.1)	66,307 (17.6)	
Smoking status, *N* (%)				< 0.001
Never	238,470 (54.8)	31,480 (53.7)	206,990 (55.0)	
Previous	151,644 (34.9)	21,250 (36.2)	130,394 (34.6)	
Current	44,926 (10.3)	5,905 (10.1)	39,021 (10.4)	
BMI (kg/m^2^), Mean (*SD*)	27.41 (4.78)	28.26 (5.37)	27.28 (4.67)	< 0.001
BMI category, *N* (%)				< 0.001
Normal (<25)	144,476 (33.2)	16,885 (28.8)	127,591 (33.9)	
Overweight (25∼30)	184,782 (42.5)	23,863 (40.7)	160,919 (42.8)	
Obesity (30∼)	105,782 (24.3)	17,887 (30.5)	87,895 (23.4)	
Vitamin D concentrations (nmol/L), Mean (*SD*)	48.32 (19.66)	47.03 (19.52)	48.52 (19.67)	< 0.001
Vitamin D concentrations (nmol/L)^a^, *N* (%)				< 0.001
12.8–32.6	108,839 (25.0)	15,966 (27.2)	92,873 (24.7)	
32.7–46.8	109,258 (25.1)	14,993 (25.6)	94,265 (25.0)	
46.9–62.0	108,744 (25.0)	14,279 (24.4)	94,465 (25.1)	
62.1–104.0	108199 (24.9)	13397 (22.8)	94802 (25.2)	
Vitamin D category (nmol/L), *N* (%)				< 0.001
Deficient (<25)	55,438 (12.7)	8,378 (14.3)	47,060 (12.5)	
Insufficient (25∼50)	186,302 (42.8)	25,697 (43.8)	160,605 (42.7)	
Optimal (>50)	193,300 (44.4)	24,560 (41.9)	168,740 (44.8)	
Vitamin D supplements, *N* (%)	7,899 (1.8)	1,124 (1.9)	6,775 (1.8)	0.048
FEV1 (mL), Mean (*SD*)	2,829 (800)	2,596 (804)	3,562 (1,107)	< 0.001
FVC (mL), Mean (*SD*)	3,735 (1,063)	2,862 (793)	3,760 (1,054)	< 0.001
FEV1/FVC (%), Mean (*SD*)	75.9 (7.3)	73.0 (8.6)	76.3 (7.0)	< 0.001
Current wheeze, *N* (%)		36,204 (61.7)		
At least one asthma hospitalization^b^, *N* (%)		29,496 (50.3)		

^a^These data were divided by quartiles.

^b^Hospitalization for asthma was defined as ever having had a hospitalization with a main diagnosis compatible with asthma, excluding hospitalizations with a main diagnosis consistent with COPD.

The relationship between circulating vitamin D level and asthma prevalence was estimated and summarized in Table 2. The results showed that per SD increase in vitamin D level was associated with 4.0% decreased odds ratio of asthma [adjusted odds ratio (*aOR*) = 0.960; 95% *CI*: 0.951–0.970; *p* < 0.001] after fully adjusting sex, age, ethnicity, BMI, income, smoking status, education, and vitamin D supplements. Then, the participants were divided into four parts by quartiles of serum vitamin D concentrations and three categories by clinical cut-offs. Participants whose vitamin D level was above the first quartile (Q1) had 4.2–9.1% significantly lower prevalence of asthma than those with levels at the first quartile in adjusted model. Moreover, compared to vitamin D deficiency, the odds of asthma were decreased by 6.4% (*aOR* = 0.936; 95% *CI*: 0.911–0.962; *p* < 0.001) and 9.8% (*aOR* = 0.902; 95% *CI*: 0.877–0. 0.927; *p* < 0.001) in individuals with insufficient and optimal vitamin D concentration, respectively, in the fully adjusted model.

**TABLE 2:** Association of vitamin D concentrations with the risk of asthma.

Vitamin D concentration, nmol/L	Total (*N* = 435,040)			Female (*N* = 233,393)			Male (*N* = 201,647)		
			
	Asthma *N* (%)	OR (95%CI)	*P*	Asthma *N* (%)	OR (95%CI)	*P*	Asthma *N* (%)	OR (95%CI)	*P*
Per *SD* in concentration	58,635 (13.5)	0.960 (0.951–0.970)	<0.001	33,371 (14.3)	0.970 (0.957–0.983)	<0.001	25,264 (12.5)	0.949 (0.935–0.963)	<0.001
Quartiles									
12.8–32.6	15,966 (14.7)	Ref		9,125 (15.7)	Ref		6,841 (13.5)	Ref	
32.7–46.8	14,993 (13.7)	0.958 (0.935–0.981)	<0.001	8,515 (14.5)	0.964 (0.933–0.996)	0.027	6,478 (12.8)	0.953 (0.919–0.989)	0.011
46.9–62.0	14,279 (13.1)	0.939 (0.916–0.962)	<0.001	8,103 (13.9)	0.956 (0.925–0.988)	0.008	6,176 (12.2)	0.921 (0.887–0.956)	<0.001
62.1–104.0	13,397 (12.4)	0.909 (0.887–0.933)	<0.001	7,628 (13.0)	0.931 (0.900–0.963)	<0.001	5,769 (11.6)	0.887 (0.853–0.921)	<0.001
		P_trend_<0.001			P_trend_ = 0.001			P_trend_ < 0.001	
Category									
Deficient (< 25)	8,378 (15.1)	Ref		4,802 (16.3)	Ref		3,576 (13.8)	Ref	
Insufficient (25–50)	25,697 (13.8)	0.936 (0.911–0.962)	<0.001	14,607 (14.7)	0.944 (0.911–0.979)	0.002	11,090 (12.8)	0.931 (0.894–0.970)	0.001
Optimal (> 50)	24,560 (12.7)	0.902 (0.877–0.927)	<0.001	13,962 (13.4)	0.922 (0.889–0.957)	<0.001	10,598 (11.9)	0.881 (0.845–0.919)	<0.001
		P_trend_<0.001			P_trend_ < 0.001			P_trend_ < 0.001	

All models were adjusted for age, sex, BMI, ethnicity, income, education, smoking status, and vitamin D supplements.

**FIGURE 2:**
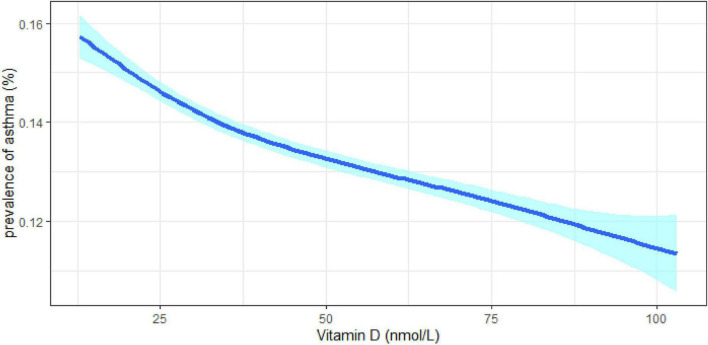
Prevalence of asthma by serum levels of vitamin D.

**FIGURE 3:**
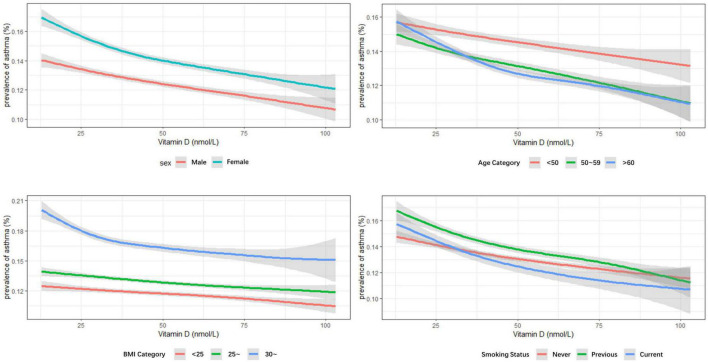
Multivariable analysis of serum vitamin D levels and prevalence of asthma stratified by sex, age groups, body mass index (BMI) categories, and smoking status.

Subgroup analysis was on the basis of sex (Table 2), age groups (Supplementary Table 1), BMI categories (Supplementary Table 2), and smoking status (Supplementary Table 3). Similar results were obtained in both men and women; the optimal vitamin D level had 7.8% lower odds of asthma in women and 11.9% in men in contrast to deficient vitamin level. The largest effect size was observed in the population older than 60 years old based on age categories. And in the BMI stratified analysis, the effect seemed more obvious in obese participants (BMI > 30 kg/m^2^). Compared with different smoking statuses, current smokers and previous smokers revealed larger estimated effects. When stratified by sex, age groups, BMI categories, and smoking status, the results were quite similar, although some of the associations had no statistical significance. Consistent results were obtained in the sensitivity analysis that all eligible participants with missing data (*n* = 438,795) were included (Supplementary Table 4). The prevalence of asthma decreased with increasing vitamin D concentrations, irrelevant to gender, age, BMI, and smoking status (*p*_trend_ < 0.05) (Figures 2, 3).

Multivariable analysis was conducted to determine the association between vitamin D concentrations and lung function among asthmatic patients (Table 3). Among total asthma patients, each nmol/L increase in vitamin D was connected with an increase of 1–2 ml in FEV1 or FVC significantly. When stratified by sex, each nmol/L increase in vitamin D was significantly linked to 1.308 ml increase in FEV1 of women, 1.987 and 2.149 ml increase in FVC of women and men, respectively. Meanwhile, increasing each nmol/L in vitamin D was along with an increase of 0.6% in the ratio of FEV1 to FVC. The analysis between vitamin D concentrations and current wheeze or asthma hospitalization was presented in Table 4. The results showed that participants with asthma whose vitamin D level was in the deficient category had 9.3–19.9% higher odds of current wheeze than insufficient categories (*aOR* = 0.907; 95% *CI*: 0.861–0.957; *p* < 0.001) and optimal categories (*aOR* = 0.801; 95% *CI*: 0.759–0.845; *p* < 0.001). However, the association of vitamin D and have ever had at least one asthma hospitalization had no statistical significance.

**TABLE 3:** Multivariable analysis of vitamin D concentrations and lung function measures among asthmatic patients.

Vitamin D concentration, Nmol/L	Total asthma patients (*N* = 58,635)		Female (*N* = 33,371)		Male (*N* = 25,264)	
			
	β (95% CI)	*P*	β (95% CI)	*P*	β (95% CI)	*P*
FEV1 (mL)	1.328 (0.575–2.080)	0.001	1.308 (0.526–2.090)	0.001	1.090 (−0.190–2.369)	0.095
FVC (mL)	2.018 (1.127–2.908)	<0.001	1.987 (1.039–2.936)	<0.001	2.149 (0.637–3.661)	0.005
FEV1/FVC (%)	0.006 (0.002–0.010)	0.003	0.006 (0.002–0.010)	0.003	0.006 (0.000–0.012)	0.056

All models were adjusted for age, sex, BMI, ethnicity, income, education, smoking status, and vitamin D supplements. Models for FEV1 and FVC were additionally adjusted for height and height squared.

**TABLE 4:** Association of vitamin D concentrations with risk of wheeze and hospitalization among asthma patients.

Vitamin D concentration, nmol/L	Current wheeze			At least one asthma hospitalization		
		
	*N* (%)	OR (95%CI)	*P*	*N* (%)	OR (95%CI)	*P*
Per *SD* in concentration	36,204 (61.7)	0.914 (0.897–0.931)	<0.001	29,496 (50.3)	1.018 (0.999–1.036)	0.063
**Quartiles**						
12.8–32.6	10,509 (65.8)	Ref		8,037 (50.3)	Ref	
32.7–46.8	9,361 (62.4)	0.909 (0.867–0.953)	<0.001	7,516 (50.1)	1.007 (0.963–1.055)	0.748
46.9–62.0	8,604 (60.3)	0.859 (0.819–0.901)	<0.001	7,213 (50.5)	1.038 (0.991–1.088)	0.115
62.1–104.0	7,730 (57.7)	0.799 (0.761–0.839)	<0.001	6,730 (50.2)	1.050 (1.000–1.101)	0.048
		P_trend_ < 0.001			P_trend_ = 0.143	
**Category**						
Deficient (< 25)	5,597 (66.8)	Ref		4,220 (50.4)	Ref	
Insufficient (25∼50)	16,217 (63.1)	0.907 (0.861–0.957)	<0.001	12,977 (50.5)	1.020 (0.970–1.073)	0.444
Optimal (>50)	14,390 (58.6)	0.801 (0.759–0.845)	<0.001	12,299 (50.1)	1.034 (0.982–1.089)	0.202
		P_trend_ < 0.001			P_trend_ = 0.424	

All models were adjusted for age, sex, BMI, ethnicity, income, education, smoking status, and vitamin D supplements.

## Discussion

In the current study, we found a strong association, independent of sex, age groups, BMI categories, and smoking status, between higher peripheral serum vitamin D level and lower odds of asthma and current wheeze among a large cohort study from the UK biobank. Moreover, asthmatic patients with higher serum vitamin D levels were more likely to have better lung function of FEV1, FVC, and FEV1/FVC.

Numerous studies tried to find out the association between circulating vitamin D level and the prevalence of asthma, getting inconclusive results. The previous studies found that vitamin D level was significantly lower with a higher prevalence of vitamin D deficiency in asthmatic patients compared with control participants (22, 23). Several cross-sectional surveys, including 6,857 US participants, reported consistent results with us, that each 10 ng/ml decrease in vitamin D level was linked with an increased prevalence of asthma (13). Other studies also found that vitamin D deficiency was along with increased odds of current asthma (24, 25). A different result showed that the prevalence of vitamin D deficiency was higher in participants with asthma but had no statistical relationship with asthma for women in a case–control study (14). However, the participants were limited in numbers and lack of the representation of men. A previous study of 307,900 Israeli adults between age 22 and 50 reported that no significant link exists between vitamin D deficiency and asthma prevalence (17), which was consistent with a meta-analysis that included 23 studies (26). Even in subjects with abundant solar exposure, vitamin D deficiency has been documented. This is likely due to a combination of behavioral factors (e.g., clothing coverage, increased time spent indoors, and sunscreen use) and intrinsic factors such as increased cutaneous destruction of vitamin D3, decreased cutaneous production of vitamin D3, or skin melanin content (27). Summarily, the reason for the difference among various studies may be the feature of study cohorts. In our finding, circulating vitamin D concentrations were inversely associated with asthma prevalence and the effect seems to be larger in old, obese individuals, and current or previous smokers, but regardless of different sex. The roles of age, smoking status, or BMI played in association between vitamin D and asthma were seldom discussed and unclear but the result may indicate their effect. In our study, the asthmatic group was more obese, more ever, and current smokers. The greater protective effect size of obese participants could be the situation that asthma is relatively common and difficult to be treated in older adults, especially in persons older than 65 (28). The prevalence of vitamin D deficiency was higher in obese subjects and there was a significant inverse link between BMI and serum vitamin D levels (29, 30). Meanwhile, being overweight increases the possibility of having or developing asthma (31). This association may explain the results that the larger effect size of vitamin D on the prevalence of asthma in the obese group. In a prospective cohort of 46,182 US black women with 16 years of follow-up, former active smoker, current active smoker, and passive smoker compared to never active/passive smoker, increased the risk of adult-onset asthma and the association was more pronounced in obese and older population (32). To some extent, these studies could give some explanations to the inconsistent effect size in stratified analyses.

Our findings corroborate most of the prior studies of vitamin D and lung function. A meta-analysis including 27 studies and another meta-analysis of a total of 14 randomized controlled trials ultimately indicated the positive association between vitamin D and lung function (18, 33). A similar association was found in a cross-sectional study of 560 children, another study of 10,860 children and 24,115 adults, and a clinical trial including a total of 54 adult participants (24, 30, 34). After analyzing data on vitamin D and lung function in asthmatic adults with small sample size, a weak positive association was observed in a prospective cohort study, being not statistically significant (35). A randomized controlled trial demonstrated that alfacalcidol, a vitamin D hormone analog, contributed to markedly increases in FEV1 and FVC in adult asthmatic patients, especially in patients with severe asthma (23).

The link between vitamin D and asthma control has been commonly discussed in the previous studies; however, the relationship was uncertain. The possible explanations of vitamin D being associated with asthma development were altering the infiltration of immune cells and inflammatory response, affecting airway remodeling through airway smooth muscle cell proliferation and secretion of fibrotic mediators, etc. (5, 36, 37). It was indicated that lower vitamin D was linked with greater odds of current wheeze in some studies (13, 24). The case–control studies reported that the severity of vitamin D deficiency is associated with poor control of asthma (14, 17). Vitamin D supplementation ameliorated asthma control, such as requiring emergency department attendance, hospitalization, number of asthma attacks, and so on (19, 38). But recently, randomized controlled clinical trials of children and adults showed that vitamin D supplementation did not have significant effect on alleviating asthma exacerbation (39, 40). A study with a relatively small sample size of asthmatic patients found that omalizumab treatment could induce a significant increase in the 25(OH)D levels and Asthma Control Test (ACT) scores compared to pre-treatment levels (41), which may indicate the association between vitamin D and asthma control. Our observational study found that the serum vitamin D had a negative relationship with current wheeze but was not associated with asthma hospitalization, which may suggest that the serum vitamin D level was possible to influence the control of asthma. The data of current wheeze represented the current control while the data of asthma hospitalization indicated how the disease had been controlled up to now. The reason why there was no significant association between vitamin D and having ever had at least one asthma hospitalization may be the uncertainty of time that contributed to the uncertainty of the current control status of disease. Thus, combining our findings and previous studies, we deliberately conclude that vitamin D may be useful prevention of asthma prevalence and disease control (5, 42). While a recommendation for supplying vitamin D as a preventive therapy, the absolute frequency and amount, for all asthma patients cannot be made due to the known limitations.

The strength of the study includes the large sample size and the potential to adjust for potential confounders such as sex, age, BMI categories, and smoking status. Nevertheless, this study also has several drawbacks. First, in a cross-sectional study, a causal relationship cannot be confirmed due to the lack of temporal relationships. Second, the age distribution of this study varied from 37 to 73 and the population younger or older lacks the estimation. Third, the possibility of recall bias and misclassification of asthma that existed due to the disease was defined using self-reported information, such as diagnosis of asthma, ever hospitalization for asthma, current wheeze in the last year, etc., depending on the participants’ ability to recall. First, we could not preclude the possibility of a large sample size making little difference meaningful; hence, the interpretation of results should be cautious. Last but not the least, the participants with missing data on crucial covariates were excluded, while the sensitivity analysis verified the findings with similar results after multiple imputation for missing covariates.

Collectively, our study found that the higher serum vitamin D levels were associated with lower odds of asthma and current wheeze was in British adults. In our results, higher vitamin D level also has positive effects on lung function. The results indicate a beneficial correlation between vitamin D and asthma, but longitudinal studies containing all age groups were necessary to provide more convincing evidence.

## Data availability statement

Publicly available datasets were analyzed in this study. This data can be found here: https://www.ukbiobank.ac.uk/.

## Ethics statement

The studies involving human participants were reviewed and approved by the North West Multi-Center Research Ethics Committee (MREC). The patients/participants provided their written informed consent to participate in this study.

## Author contributions

All authors listed have made a substantial, direct, and intellectual contribution to the work, and approved it for publication.

## Footnotes

1
https://www.ukbiobank.ac.uk/
