# Supplementary material for: Vitamin D status and asthma, lung function, and hospitalization among British adults
Source: Front Nutr. 2022 Aug 10;9:954768. doi: 10.3389/fnut.2022.954768 (PMC9399919; doi:10.3389/fnut.2022.954768)
Supplement: Supplementary file 1 [file Data_Sheet_1.docx]

eTable 1. Association of vitamin D concentrations with asthma in different age categories.

| vitamin D concentration,  nmol/L | <50 years old  (N=102949) | | |  | 50-59 years old  (N=144179) | | |  | >60 years old  (N=187912) | | |
| --- | --- | --- | --- | --- | --- | --- | --- | --- | --- | --- | --- |
|  | Asthma  N (%) | OR (95%CI) | *P* |  | Asthma  N (%) | OR (95%CI) | *P* |  | Asthma  N (%) | OR (95%CI) | *P* |
| Per SD in concentration | 15015 (14.7) | 0.978 (0.960-0.997) | 0.024 |  | 19133 (13.3) | 0.962 (0.946-0.979) | <0.001 |  | 24397 (13.0) | 0.948 (0.933-0.962) | <0.001 |
| Quartiles |  |  |  |  |  |  |  |  |  |  |  |
| 12.8-32.6 | 4792 (15.3) | Ref |  |  | 5475 (14.2) | Ref |  |  | 5699 (14.6) | Ref |  |
| 32.7-46.8 | 3926 (14.8) | 0.976 (0.932-1.023) | 0.311 |  | 4991 (13.5) | 0.977 (0.937-1.019) | 0.278 |  | 6076 (13.2) | 0.922 (0.886-0.959) | <0.001 |
| 46.9-62.0 | 3359 (14.5) | 0.976 (0.929-0.124) | 0.316 |  | 4557 (13.0) | 0.959 (0.919-1.001) | 0.058 |  | 6363 (12.6) | 0.895 (0.861-0.931) | <0.001 |
| 62.1-104.0 | 3028 (13.8) | 0.952 (0.905-1.001) | 0.055 |  | 4110 (12.2) | 0.921 (0.881-0.963) | <0.001 |  | 6259 (11.9) | 0.872 (0.838-0.907) | <0.001 |
|  |  | P_trend_=0.290 |  |  |  | P_trend_=0.003 |  |  |  | P_trend_<0.001 |  |
| Category |  |  |  |  |  |  |  |  |  |  |  |
| Deficient (<25) | 2603 (15.5) | Ref |  |  | 2951 (14.8) | Ref |  |  | 2824 (15.1) | Ref |  |
| Insufficient (25~50) | 6856 (14.8) | 0.971 (0.925-1.021) | 0.252 |  | 8564 (13.5) | 0.930 (0.889-0.974) | 0.002 |  | 10277 (13.4) | 0.906 (0.866-0.948) | <0.001 |
| Optimal (>50) | 5646 (14.2) | 0.961 (0.913-1.012) | 0.135 |  | 7618 (12.5) | 0.897 (0.856-0.940) | <0.001 |  | 11296 (12.2) | 0.861 (0.823-0.901) | <0.001 |
|  |  | P_trend_=0.325 |  |  |  | P_trend_<0.001 |  |  |  | P_trend_<0.001 |  |

All models were adjusted for sex, BMI, ethnicity, income, education, smoking status, and vitamin D supplements.

eTable 2. Association of vitamin D concentrations with asthma in different BMI categories.

| vitamin D concentration,  nmol/L | <25  (N=144476) | | |  | 25~  (N=184782) | | |  | 30~  (N=105782) | | |
| --- | --- | --- | --- | --- | --- | --- | --- | --- | --- | --- | --- |
|  | Asthma  N (%) | OR (95%CI) | *P* |  | Asthma  N (%) | OR (95%CI) | *P* |  | Asthma  N (%) | OR (95%CI) | *P* |
| Per SD in concentration | 16885 (11.7) | 0.965 (0.948-0.981) | <0.001 |  | 23863 (12.9) | 0.961 (0.946-0.975) | <0.001 |  | 17887 (16.9) | 0.930 (0.913-0.948) | <0.001 |
| Quartiles |  |  |  |  |  |  |  |  |  |  |  |
| 12.8-32.6 | 3785 (12.2) | Ref |  |  | 5865 (13.6) | Ref |  |  | 6316 (18.2) | Ref |  |
| 32.7-46.8 | 3875 (11.8) | 0.975 (0.929-1.022) | 0.292 |  | 6136 (13.1) | 0.965 (0.929-1.003) | 0.073 |  | 4982 (16.8) | 0.922 (0.885-0.960) | <0.001 |
| 46.9-62.0 | 4302 (11.8) | 0.980 (0.935-1.027) | 0.403 |  | 6086 (12.6) | 0.927 (0.892-0.964) | <0.001 |  | 3891 (16.2) | 0.888 (0.850-0.929) | <0.001 |
| 62.1-104.0 | 4923 (11.2) | 0.927 (0.885-0.970) | 0.001 |  | 5776 (12.3) | 0.904 (0.869-0.940) | <0.001 |  | 2698 (15.6) | 0.859 (0.817-0.903) | <0.001 |
|  |  | P_trend_=0.006 |  |  |  | P_trend_<0.001 |  |  |  | P_trend_<0.001 |  |
| Category |  |  |  |  |  |  |  |  |  |  |  |
| Deficient (<25) | 1981 (12.6) | Ref |  |  | 2911 (13.7) | Ref |  |  | 3486 (18.9) | Ref |  |
| Insufficient (25~50) | 6520 (11.7) | 0.938 (0.888-0.990) | 0.020 |  | 10403 (13.2) | 0.963 (0.922-1.007) | 0.101 |  | 8774 (17.0) | 0.891 (0.853-0.931) | <0.001 |
| Optimal (>50) | 8384 (11.5) | 0.925 (0.877-0.975) | 0.004 |  | 10549 (12.5) | 0.911 (0.872-0.953) | <0.001 |  | 5627 (15.8) | 0.831 (0.792-0.871) | <0.001 |
|  |  | P_trend_=0.015 |  |  |  | P_trend_<0.001 |  |  |  | P_trend_<0.001 |  |

All models were adjusted for age, sex, ethnicity, income, education, smoking status, and vitamin D supplements.

eTable 3. Association of vitamin D concentrations with asthma in different smoking status.

| vitamin D concentration,  nmol/L | Never  (N=238470) | | |  | Previous  (N=151644) | | |  | Current  (N=44926) | | |
| --- | --- | --- | --- | --- | --- | --- | --- | --- | --- | --- | --- |
|  | Asthma  N (%) | OR (95%CI) | *P* |  | Asthma  N (%) | OR (95%CI) | *P* |  | Asthma  N (%) | OR (95%CI) | *P* |
| Per SD in concentration | 31480 (13.2) | 0.978 (0.965-0.991) | 0.001 |  | 21250 (14.0) | 0.944 (0.929-0.960) | <0.001 |  | 5905 (13.1) | 0.913 (0.886-0.941) | <0.001 |
| Quartiles |  |  |  |  |  |  |  |  |  |  |  |
| 12.8-32.6 | 8336 (14.2) | Ref |  |  | 5320 (15.5) | Ref |  |  | 2310 (14.6) | Ref |  |
| 32.7-46.8 | 8100 (13.4) | 0.975 (0.943-1.008) | 0.139 |  | 5469 (14.4) | 0.950 (0.912-0.990) | 0.015 |  | 1424 (13.0) | 0.889 (0.827-0.955) | 0.001 |
| 46.9-62.0 | 7827 (12.9) | 0.968 (0.936-1.001) | 0.059 |  | 5288 (13.6) | 0.913 (0.875-0.952) | <0.001 |  | 1164 (12.5) | 0.871 (0.807-0.941) | <0.001 |
| 62.1-104.0 | 7217 (12.2) | 0.944 (0.912-0.978) | 0.001 |  | 5173 (12.8) | 0.881 (0.845-0.920) | <0.001 |  | 1007 (11.4) | 0.807 (0.744-0.875) | <0.001 |
|  |  | P_trend_=0.015 |  |  |  | P_trend_<0.001 |  |  |  | P_trend_<0.001 |  |
| Category |  |  |  |  |  |  |  |  |  |  |  |
| Deficient (<25) | 4225 (14.3) | Ref |  |  | 2749 (16.3) | Ref |  |  | 1404 (15.4) | Ref |  |
| Insufficient (25~50) | 13877 (13.5) | 0.982 (0.946-1.019) | 0.335 |  | 9207 (14.4) | 0.904 (0.862-0.947) | <0.001 |  | 2613 (13.2) | 0.847 (0.789-0.909) | <0.001 |
| Optimal (>50) | 13378 (12.6) | 0.963 (0.927-1.001) | 0.054 |  | 9294 (13.1) | 0.852 (0.813-0.893) | <0.001 |  | 1888 (11.8) | 0.780 (0.723-0.841) | <0.001 |
|  |  | P_trend_=0.114 |  |  |  | P_trend_<0.001 |  |  |  | P_trend_<0.001 |  |

All models were adjusted for age, sex, BMI, ethnicity, income, education, and vitamin D supplements.

eTable 4. Sensitivity analysis (using multiple imputation for missing covariates) of vitamin D concentration and asthma in all eligible participants.

| vitamin D concentration | Total  (N=438795) | | |  | Female  (N=235281) | | |  | Male  (N=203514) | | |
| --- | --- | --- | --- | --- | --- | --- | --- | --- | --- | --- | --- |
|  | Asthma  N (%) | OR (95%CI) | *P* |  | Asthma  N (%) | OR (95%CI) | *P* |  | Asthma  N (%) | OR (95%CI) | *P* |
| Per SD in concentration | 59211 (13.5) | 0.965 (0.956-0.974) | <0.001 |  | 33671 (14.3) | 0.975 (0.963-0.986) | <0.001 |  | 25540 (12.5) | 0.955 (0.942-0.968) | <0.001 |
| Quartiles |  |  |  |  |  |  |  |  |  |  |  |
| 12.8-32.6 | 16209 (14.7) | Ref |  |  | 9236 (15.7) | Ref |  |  | 6973 (13.5) | Ref |  |
| 32.7-46.8 | 15015 (13.7) | 0.964 (0.941-0.988) | 0.003 |  | 8518 (14.5) | 0.965 (0.934-0.997) | 0.032 |  | 6497 (12.8) | 0.966 (0.931-1.002) | 0.062 |
| 46.9-62.0 | 14426 (13.2) | 0.947 (0.924-0.971) | <0.001 |  | 8187 (14.0) | 0.963 (0.931-0.995) | 0.025 |  | 6239 (12.2) | 0.930 (0.896-0.965) | <0.001 |
| 62.1-104.0 | 13561 (12.4) | 0.916 (0.893-0.939) | <0.001 |  | 7730 (13.0) | 0.936 (0.905-0.969) | <0.001 |  | 5831 (11.6) | 0.894 (0.860-0.929) | <0.001 |
|  |  | P_trend_<0.001 |  |  |  | P_trend_=0.002 |  |  |  | P_trend_<0.001 |  |
| Category |  |  |  |  |  |  |  |  |  |  |  |
| Deficient (<25) | 8438 (15.2) | Ref |  |  | 4824 (16.3) | Ref |  |  | 3614 (13.9) | Ref |  |
| Insufficient (25~50) | 25926 (13.8) | 0.937 (0.911-0.962) | <0.001 |  | 14714 (14.7) | 0.942 (0.909-0.977) | 0.001 |  | 11212 (12.8) | 0.932 (0.895-0.972) | 0.001 |
| Optimal (>50) | 24847 (12.7) | 0.906 (0.881-0.932) | <0.001 |  | 14133 (13.4) | 0.927 (0.893-0.961) | <0.001 |  | 10714 (11.9) | 0.885 (0.849-0.923) | <0.001 |
|  |  | P_trend_<0.001 |  |  |  | P_trend_<0.001 |  |  |  | P_trend_<0.001 |  |

All models were adjusted for age, sex, BMI, ethnicity, income, education, smoking status, and vitamin D supplements.
